# A new approach to measure dwell position inaccuracy in HDR ring applicators – quantification and corrective QA

**DOI:** 10.1120/jacmp.v12i1.3355

**Published:** 2010-09-09

**Authors:** Abdul Qadir Jangda, Sherali Hussein, Zaka Rehman

**Affiliations:** ^1^ Department of Radiation Oncology The Aga Khan University Hospital Karachi Pakistan

**Keywords:** high dose rate brachytherapy, remote afterloaders, ring applicators, quality assurance

## Abstract

As part of quality assurance (QA) in high dose rate brachytherapy, it is necessary to verify that the source dwell positions correspond to the radiographic markers used in simulation and treatment planning. The procedure is well established for linear tandem applicators. However, with the advent of ring applicators, this has become more critical and challenging. This work describes a new approach to determine positional inaccuracies for ring applicators in which the dummy markers are imaged just once and their dwell positions characterized with respect to an applicator‐defined axis. The radiograph serves as a reference for dummy markers for comparison with all subsequent measurements in which the active sources are autoradiographed at different offsets – thus obviating the back‐and‐forth transferring of setup between afterloader and simulator. The method has been used specifically to characterize the Varian GammaMed 60° ring applicator, but it may be adapted to any other applicator. The results show that an offset of 1–2 mm minimizes the overall inaccuracy to within ±2 mm.

PACS numbers: 87.53.Jw, 87.56.Fc, 87.56.B, 87.56.bg, 87.56.‐v

## I. INTRODUCTION

Intracavitary brachytherapy in combination with external beam radiotherapy is a standard treatment for cancer of the cervix. For brachytherapy, the use of high dose rate (HDR) remote afterloader, with its dosimetric challenges due to high source activity and short treatment distance, requires vigilant quality assurance.^(^ ^1^ ^–^ ^5^ ^)^ One of the QA parameters that need to be verified is the dwell position accuracy, which may be defined as the correspondence between the planned dwell positions (simulated with dummy sources) and those achieved during treatment with active sources. The American Association of Physicists in Medicine Task Group No. 56^(^ ^2^ ^)^ recommends this correspondence be within ±2 mm referenced to the applicator system.

With the advent of ring applicators, the verification of dwell position accuracy has become even more critical and challenging, since the trajectory of the source wire along the circular lumen of the applicator with an inner diameter larger than source wire diameter results in so‐called “snaking” within the lumen. Varian Medical Systems^(^ ^6^ ^)^ have reported positional inaccuracies with their ring applicators that do not always meet the ±2 mm criterion. Their test data show a 2–4 mm proximal offset from the distal‐most dwell position. They recommend that each applicator be individually characterized prior to use to determine the offset correction and verified at each source change. Waid et al.^(^ ^7^ ^)^ have reported differences between the planned and treatment positions of up to 5 mm. Similarly, Stern et al.^(^ ^8^ ^)^ found the differences in the range of 2–6 mm, and used a global shift of 3 mm to correct all but the most proximal dwell positions to within ±2 mm.

A conventional method^(^ ^4^ ^)^ to verify dwell position accuracy uses kilovoltage radiography to image the dummy markers followed by autoradiography with active sources on a single film with the applicator in the same geometry. To determine the optimum offset, this requires the procedure to be repeated for a number of different offsets, ensuring reproducibility of setup between radiography and autoradiography. This work describes a new approach in which the dummy markers are imaged just once and their dwell positions characterized with respect to an applicator‐defined axis. The radiograph serves as a reference for the dummy markers for comparison with all subsequent measurements in which the active sources are autoradiographed at different offsets – thus obviating the back‐and‐forth transferring of the setup between afterloader and simulator. The method has been used specifically to characterize the Varian GammaMed 60° ring applicator, but it may be adapted to any other applicator. The results show that an offset of 1–2 mm minimizes the overall inaccuracy to within ±2 mm. For comparison, measurements were also done using the conventional method; the two sets of results are comparable within experimental error.

## II. MATERIALS AND METHODS

In the Varian GammaMed high dose rate remote afterloader unit (Varian Medical Systems, Palo Alto, CA), when the active source is transported to a tandem applicator it hits the distal end of the applicator and is retracted by 1 mm, so that its position coincides with that of the distal‐most dummy source. It is important to verify this correspondence with a tandem applicator before proceeding to any measurements with ring applicators. Indeed, this is the accepted QA procedure for tandem applicators.^(^ ^2^ ^,^ ^4^ ^)^

### A. Exposure parameters

To determine the optimum film quality, test films were exposed with different combinations of exposure parameters. The best combination in terms of contrast and brightness was a dwell time of 0.3–0.5 seconds (corresponding to a 10 Ci source) and a step size of 1.0 cm for autoradiography, and 125 ${\text{KV}}_{p}$ and 200 mAs for radiography. It was also found that the film quality can be enhanced significantly by placing a high‐Z material shield in the central cavity of the ring to block the cross‐exposures from dwell positions across the ring. The available eyeshield was used for this purpose.

### B. Applicator‐based reference axis

**Figure 1:**
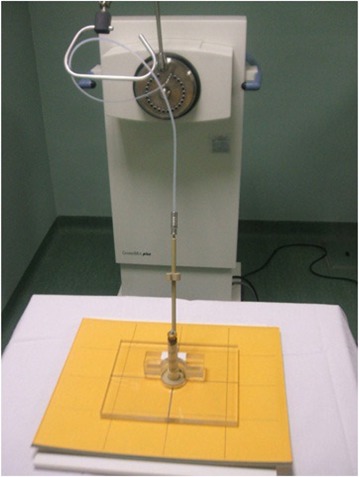
Geometrical setup showing ring applicator in Perspex jig (with the central eyeshield).

**Figure 2:**
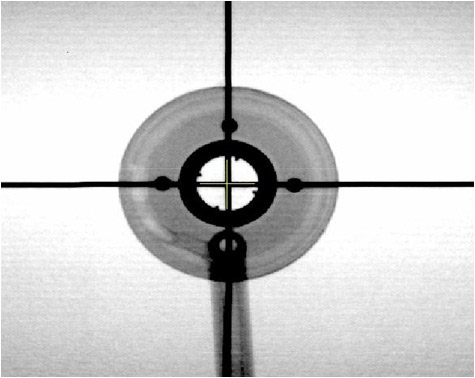
Simulation radiograph showing the four opaque markers, at right angles to each other, used to define the ring axis.

**Figure 3:**
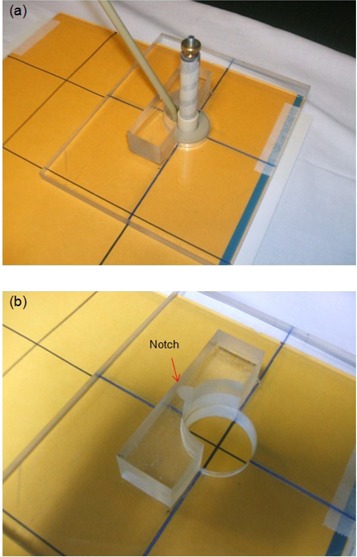
Perspex jig (a) aligned with the straight line (pinpricks) on film envelop; (b) close‐up view with applicator removed showing design details for applicator immobilization.

For the actual measurements with the Varian 60° GammaMed ring applicator (GM11000760), a custom‐fabricated Perspex jig was used to immobilize the applicator for positional reproducibility, as shown in Fig 1. There are four internal opaque markers inside the ring which are perfectly at right angles to each other (Fig. 2). These markers were used to define the axis of the ring applicator. The applicator fits in the jig in a unique orientation such that the ring axis is aligned with the groove etched on the base plate of the jig. With the jig placed on the film envelop, pinpricks at both ends of the straight line aligned with the groove provide the reference ring axis on the film, as shown in Fig. 3.

### C. Measurements and data analysis

**Figure 4:**
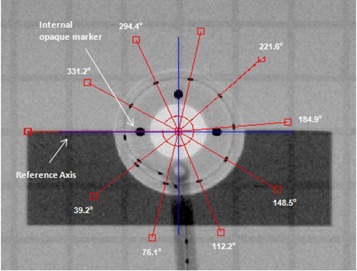
Reference radiograph with X‐ray marker showing angles measured with respect to reference axis (blue line) aligned with the four opaque markers.

With the HDR afterloader, the treatment is delivered with the active source at a number of dwell positions along the applicator. Because of this, an optimum offset needs to be determined to correct all dwell positions for a particular applicator. With the ring applicator in the jig, a simulator radiograph with the X‐ray marker in place is acquired. This serves as the reference radiograph and provides dwell positions for the dummy markers for comparison with all subsequent measurements with active sources (Fig. 4). Next, an autoradiograph is acquired with nine exposures using offsets ranging from 0 to 8 mm in 1 mm steps, all on a single film. The film envelop is marked (pinpricked) with straight lines aligned with the groove etched on the Perspex jig which, in turn, is aligned with the ring axis. The placement of the jig during consecutive exposures follows these lines and, hence, the common applicator‐based reference axis.

**Figure 5:**
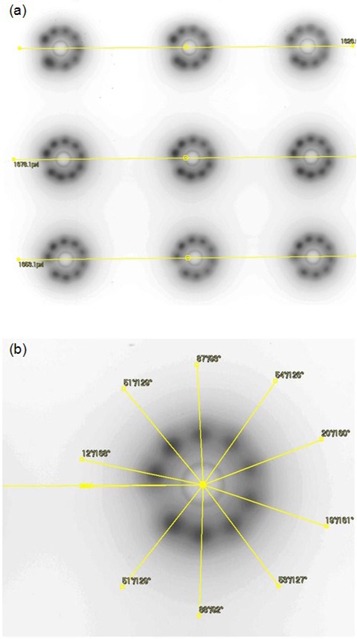
Autoradiograph (a) showing nine exposures at offsets from 0–8 mm in 1 mm steps, on a single film; (b) details showing measured angles referenced to the horizontal axis of the ring.

To analyze and determine the inaccuracies in the dwell positions, the films were first digitized with VIDAR scanner (VIDAR Systems Corp., Herndon, VA) and then imported into a K‐PACS^(^ ^9^ ^)^ workstation V1.6. The angles with respect to the axis were measured for each of the nine source dwell positions on the autoradiograph (Fig. 5) and compared with the corresponding angles of the dummy markers measured on the reference radiograph. The deviation between the two for each position was determined and converted to the corresponding path length deviation using the following equation:

(1) $$\text{Path}\;\text{length},\;L=\pi \times r\times (\alpha /180^{0})$$

where r= the ring radius (15 mm), and α= the angle measured from the reference horizontal axis of the ring. At each offset, the average deviation between the two sets of path lengths was determined.

**Figure 6:**
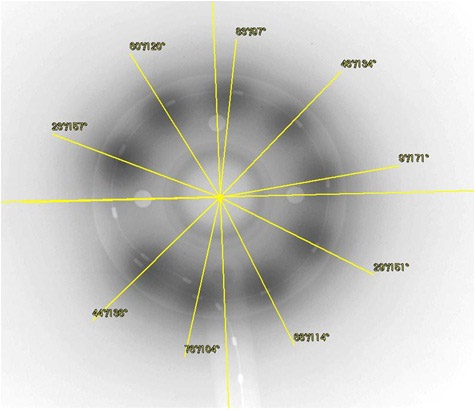
Conventional method: autoradiography and radiography on a single film for a 2 mm offset; film processed with K‐PACS software to measure the angles.

To assess our method against the conventional one,^(^ ^4^ ^)^ an autoradiograph was acquired with the nine dwell source positions, ensuring that the applicator axis remained aligned between the exposures. The setup was then moved to the simulator to acquire a radiograph with the X‐ray marker in place, as shown in Fig. 6. The experiment was repeated five times to cover the offsets range from 0 to 4 mm in 1 mm steps.

## III. RESULTS

**Figure 7:**
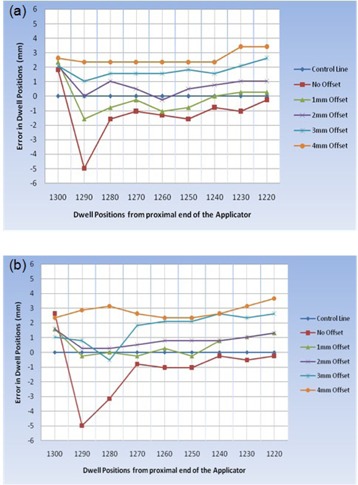
Deviation between planned and delivered dwell positions: (a) the current work; (b) conventional method.

The data analysis for the offsets of 0, 1, 2, 3 and 4 mm for the two methods are shown in Tables 1 and 2. The results for offsets ranging from 0–4 mm in steps of 1 mm are shown plotted in Fig. 7. The positive and negative deviations correspond to dwell positions that are shifted (with respect to the dummy sources) distally and proximally, respectively.

**Table 1:** Data analysis – the current work. Note that the angular spacings for X‐ray marker at each offset are identical, since they are derived from the single reference radiograph.

*Offset (mm)*	*Angular Spacing (degrees)*		*Deviation*		*Average Deviation (mm)*
	*X‐ray Marker*	*Dwell Positions*	*Angular Spacing (degrees)*	*Path Lengths (mm)*	
	39	46	7.0	1.8	
	76	57	−19.0	−5.0	
	112	106	−6.0	−1.6	
	148	144	−4.0	−1.0	
0	185	180	−5.0	−1.3	1.5
	222	216	−6.0	−1.6	
	258	255	−3.0	−0.8	
	294	290	−4.0	−1.0	
	331	330	−1.0	−0.3	
	39	48	9.0	2.4	
	76	70	−6.0	−1.6	
	112	109	−3.0	−0.8	
	148	147	−1.0	−0.3	
1	185	181	−4.0	−1.0	0.7
	222	219	−3.0	−0.8	
	258	258	0.0	0.0	
	294	295	1.0	0.3	
	331	332	1.0	0.3	
	39	47	8.0	2.1	
	76	76	0.0	0.0	
	112	116	4.0	1.0	
	148	150	2.0	0.5	
2	185	184	−1.0	−0.3	0.8
	222	224	2.0	0.5	
	258	261	3.0	0.8	
	294	298	4.0	1.0	
	331	335	4.0	1.0	
	39	47	8.0	2.1	
	76	80	4.0	1.0	
	112	118	6.0	1.6	
	148	154	6.0	1.6	
3	185	191	6.0	1.6	1.6
	222	229	7.0	1.8	
	258	264	6.0	1.6	
	294	302	8.0	2.1	
	331	341	10.0	2.6	
	39	49	10.0	2.6	
	76	85	9.0	2.4	
	112	121	9.0	2.4	
	148	157	9.0	2.4	
4	185	194	9.0	2.4	2.4
	222	231	9.0	2.4	
	258	267	9.0	2.4	
	294	77.2	307	80.4	
	331	86.7	345	90.3	

**Table 2:** Data analysis – conventional method.

*Offset (mm)*	*Angular Spacing (degrees)*		*Deviation*		*Average Deviation (mm)*
	*X‐ray Marker*	*Dwell Positions*	*Angular Spacing (degrees)*	*Path Lengths (mm)*	
	38	48	10	2.6	
	76	57	−19	−5.0	
	113	101	−12	−3.1	
	149	146	−3	−0.8	
0	186	182	−4	−1.0	1.5
	223	219	−4	−1.0	
	259	258	−1	−0.3	
	295	293	−2	−0.5	
	331	330	−1	−0.3	
	38	44	6	1.6	
	76	75	−1	−0.3	
	113	113	0	0.0	
	150	149	−1	−0.3	
1	186	187	1	0.3	0.6
	223	222	−1	−0.3	
	259	262	3	0.8	
	295	299	4	1.0	
	331	336	5	1.3	
	38	44	6	1.6	
	75	76	1	0.3	
	113	114	1	0.3	
	149	151	2	0.5	
2	186	189	3	0.8	0.7
	223	226	3	0.8	
	260	263	3	0.8	
	296	300	4	1.0	
	332	337	5	1.3	
	38	42	4	1.0	
	76	79	3	0.8	
	113	111	−2	−0.5	
	149	156	7	1.8	
3	186	194	8	2.1	1.5
	223	231	8	2.1	
	259	269	10	2.6	
	295	304	9	2.4	
	332	342	10	2.6	
	38	47	9	2.4	
	76	87	11	2.9	
	113	125	12	3.1	
	149	159	10	2.6	
4	187	196	9	2.4	2.5
	223	232	9	2.4	
	259	269	10	2.6	
	295	307	12	3.1	
	331	345	14	3.7	

The data show that the average deviation in the dwell positions decreases, at first, as the offset increases; reaches a minimum of ~1 mm at an offset of 1–2 mm, and increases again. The optimum offset lies between 1 and 2 mm. It is noted that, except for the most distal positions, the dwell position error in this offset range is within ±2 mm.

The experimental error depends on the precision with which the applicator reference axis can be marked on the film by aligning the pinpricks with the groove on the Perspex jig. This is estimated to be ±0.2 mm. It also depends on the uncertainty in measuring the angular spacing of sources with respect to the reference axis. This can be determined within ±1° using the software, which translates to ±0.3 mm in path length. Hence, the estimated error in the deviation between the X‐ray markers and the active dwell positions is ±0.5 mm.

## IV. DISCUSSION

The positional inaccuracy measured for the ring applicator without an offset varies from 1.8 mm proximally (towards afterloader) to 5.0 mm distally (away from afterloader). The optimum corrective offset of 1–2 mm minimizes the dwell position error to within the ±2 mm. Our results agree with the previously reported findings with ring applicators, both in terms of the range and the direction of the correctional offset.^(^ ^6^ ^–^ ^8^ ^)^ However, the magnitude of optimum offset determined in this work differs with the suggested offset of 3 to 4 mm. Based on this, we agree with the Varian recommendation that each applicator be individually characterized prior to its use to determine the offset correction and verified at each source change.

Our results, using the “autoradiograph alone” approach, agree within experimental error with those obtained using the conventional method. However, the method reported in this work is simpler and less laborious and is cost effective. The reference radiograph with the dummy markers is obtained just once, and can be used for comparison in subsequent characterizations. The multiple autoradiographs are acquired on a single film; hence, alleviating the back‐and‐forth transferring of the setup between the afterloader and the simulator and the need for vigilance for reproducibility between the transfers.

Finally, in this work all measurements have been referenced to the axis of the ring. This is in compliance with TG56 recommendation^(^ ^2^ ^)^ that the correspondence between the planned and the actual dwell positions be within ±2 mm with reference to the applicator system.

## V. CONCLUSIONS

The goal of this paper was to measure the dwell position inaccuracy and quantify the corrective offset in HDR ring applicators using a new approach based on autoradiography alone. We have carried out the measurements with the Varian GammaMed 60° ring applicator to demonstrate the method and its applicability to other ring applicators. The results show that an offset of 1–2 mm minimizes the overall inaccuracy to within ±2 mm. The results are in agreement, within experimental error, with those obtained with the conventional method.

This work shows that the TG56^(^ ^2^ ^)^ recommended tolerance of ±2 mm in the dwell positions is not met with the Varian 60° GammaMed ring applicator (GM11000760). Based on our findings and those reported elsewhere in literature, we conclude that each ring applicator should be individually characterized prior to its use to determine the corrective offset and verified at each source change. Such characterization should be referenced to applicator geometry in compliance with the TG56 recommendation.

Finally, it is suggested that the clinical significance of positional inaccuracies in dwell positions using ring applicators should be explored to quantify the resultant change in dose distribution in HDR brachytherapy.

## ACKNOWLEDGEMENTS

The authors would like to thank Zubair Usman, Information Systems Department at the Aga Khan University Hospital, Karachi, Pakistan, for his help in digitizing and processing the films.
